# Supplementary material for: Disruption of Multiple Overlapping Functions Following Stepwise Inactivation of the Extended Myc Network
Source: Cells. 2022 Dec 16;11(24):4087. doi: 10.3390/cells11244087 (PMC9777503; doi:10.3390/cells11244087)
Supplement: Supplementary file 1 [file cells-11-04087-s001.zip › cells-1967310-Supplementary.pdf]

cells MDPI

Supplementary Materials for

Disruption of Multiple Overlapping Functions Following Step-Wise Inactivation of the Extended Myc Network

Huabo Wang, Taylor Stevens, Jie Lu, Merlin Airik, Rannar Airik, Edward V. Prochownik*

*Corresponding author. Email: procev@chp.edu

This PDF file includes:

Figures S1 to S20
Tables S1 to S4

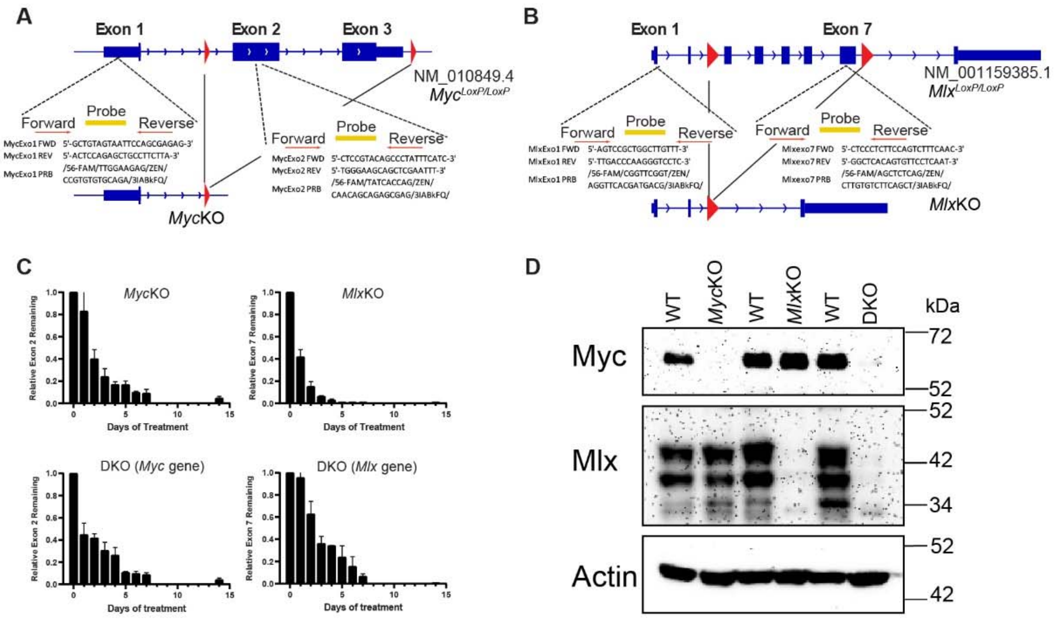

Figure S1. Time-dependent excision of *Myc* and/or *Mlx* alleles. (**A**). Structure of the "floxed" *Myc* gene in MEFs showing the position of LoxP sites flanking exons 2 and 3 and the TaqMan strategy used to quantify WT and mutant alleles (*1*). (**B**). Structure of the floxed *Mlx* gene showing the position of LoxP sites flanking exons 3 and 7 the TaqMan strategy used to quantify WT and mutant alleles (*1*). (**C**). Progressive loss of intact target gene alleles during the course of 4OHT treatment. MEFs were treated with fresh 4OHT daily (500 nM) for 7 days. DNAs were extracted from triplicate cultures and used for quantitative TaqMan-based assays to quantify the relative proportions of intact WT and KO alleles (*1*). (**D**). Immuno-blotting for Myc and Mlx proteins in the indicated cell lines performed on day 10. Similar results were obtained in 2 additional replicas (not shown). KO cells were routinely maintained in 4OHT and periodically checked for the presence of intact alleles and the expression of protein in order to ensure the stability of the KO population.

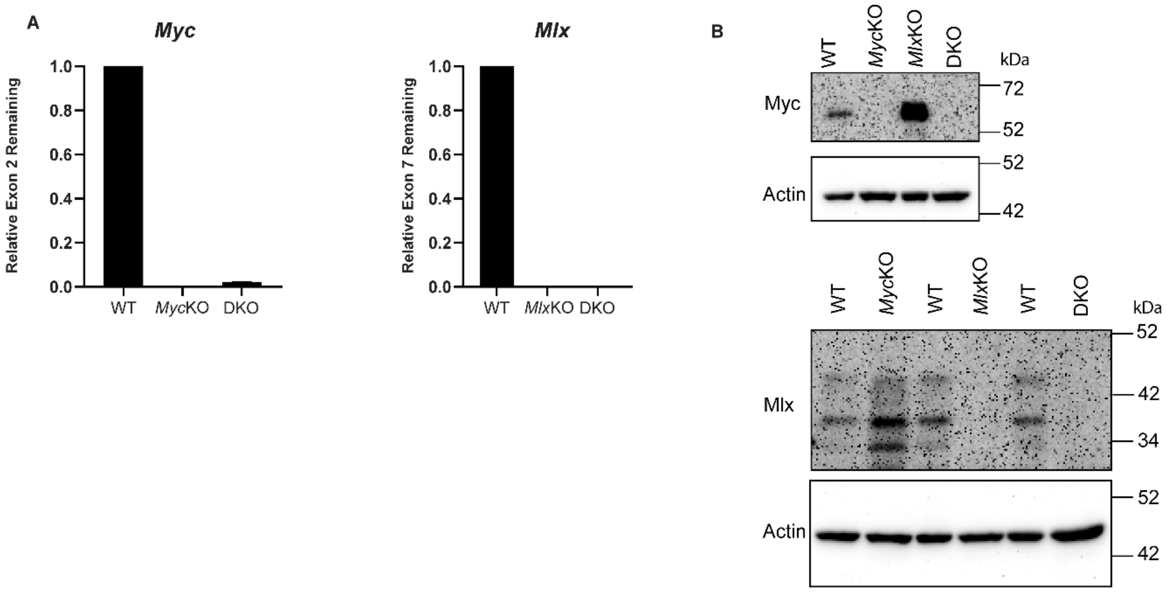

Figure S2. Excision and loss of expression of *Myc* and *Mlx* in immortalized MEFs. Early passage MEFs were transduced with an SV40 T-antigen-encoding lentiviral vector and selected in blasticidin for two weeks. Cells were then either exposed to 4OHT for 10 days or maintained in 4OHT-free medium to serve as WT control cells. (**A**). Excision of the *Myc* and/or *Mlx* loci following 10 days of 4OHT treatment. qPCR-based TaqMan assays to quantify WT and KO gene loci in each cell line were performed as described in Fig. S1A & B. **(B**). Expression of the indicated proteins detected by immuno-blotting as described in Fig. S1D.

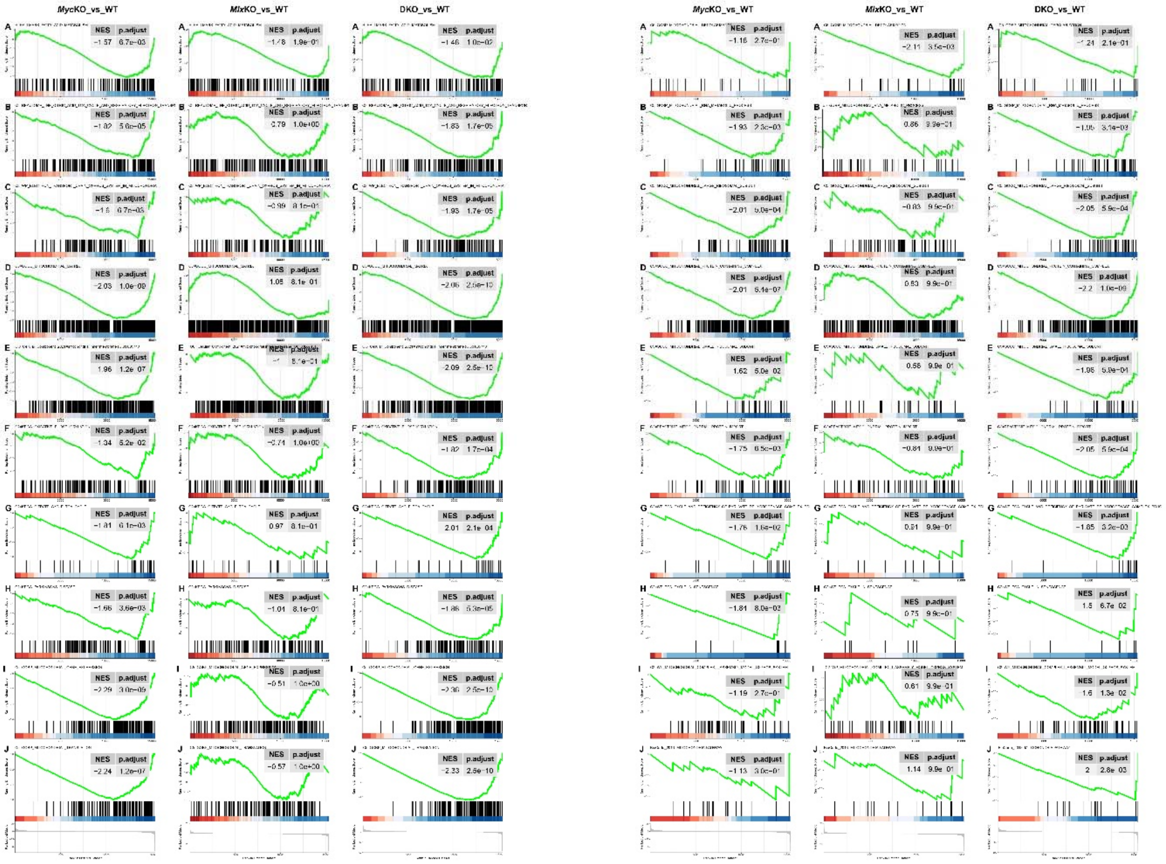

Figure S3. GSEA plots for gene sets involved in mitochondrial structure/function. Some of these gene sets are also displayed in Fig. 5D as ridgeline plots.

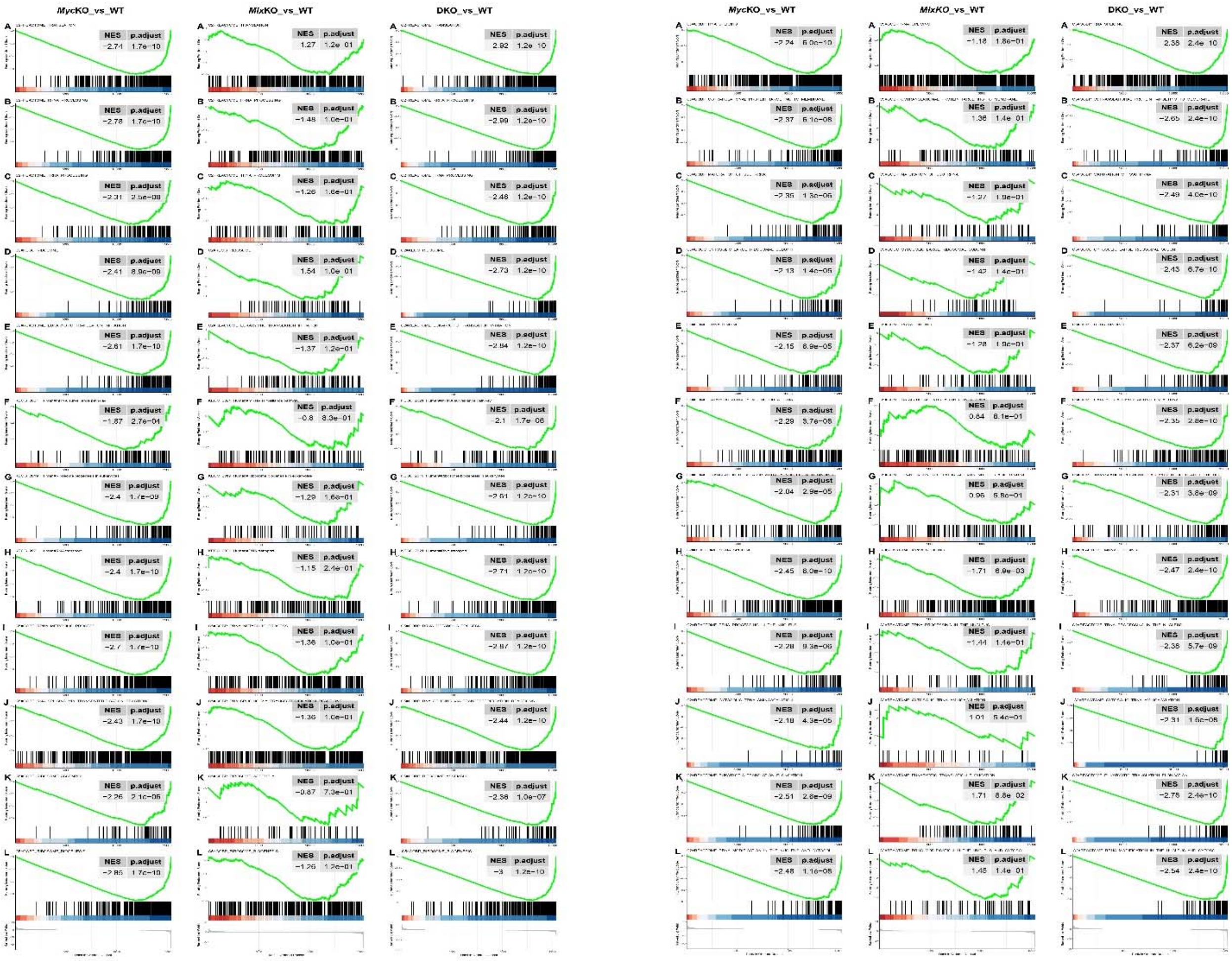

Figure S4. GSEA plots for gene sets involved in ribosome structure/translation. Some of these gene sets are also displayed in Fig. 5D as ridgeline plots.

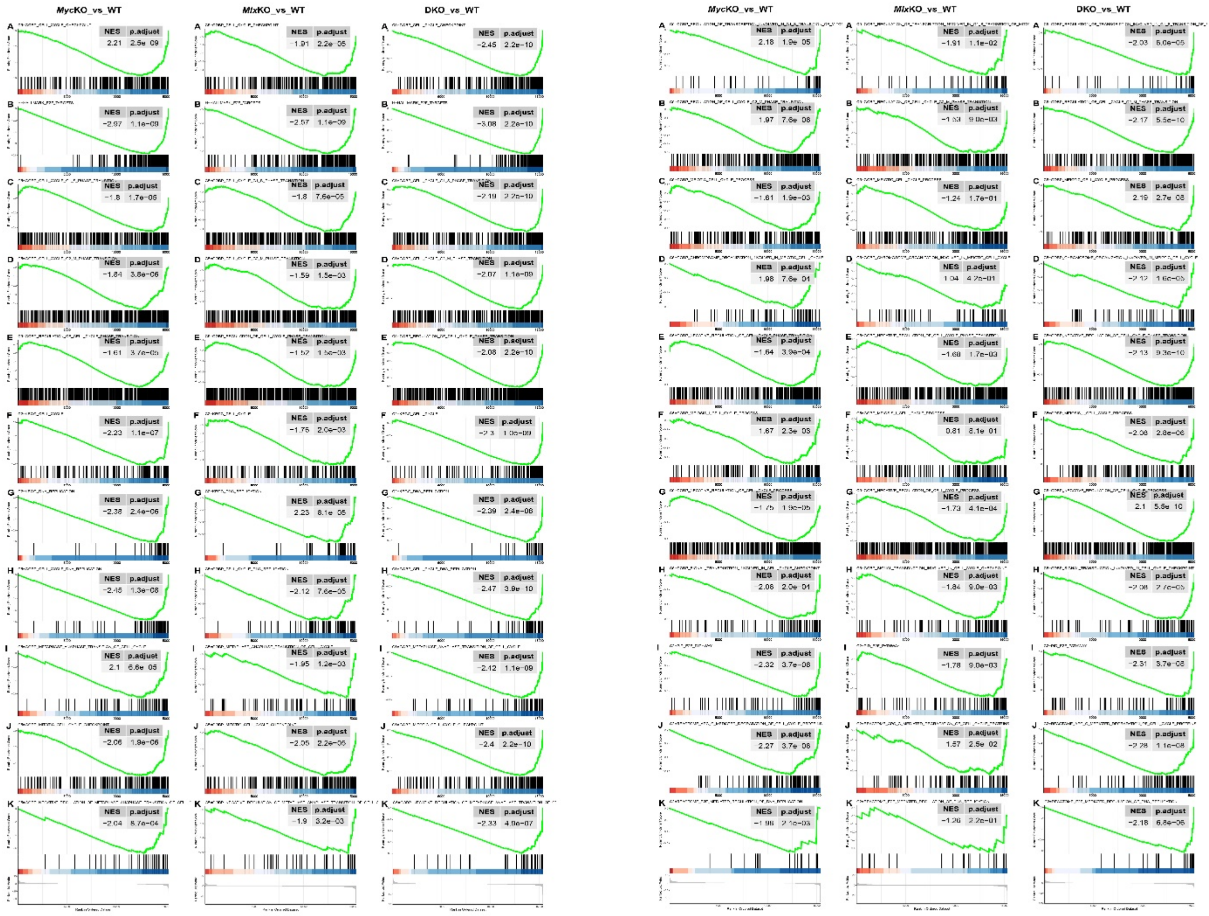

Figure S5. GSEA plots for gene sets involved in cell cycle regulation. Some of these gene sets are also displayed in Fig. 5D as ridgeline plots.

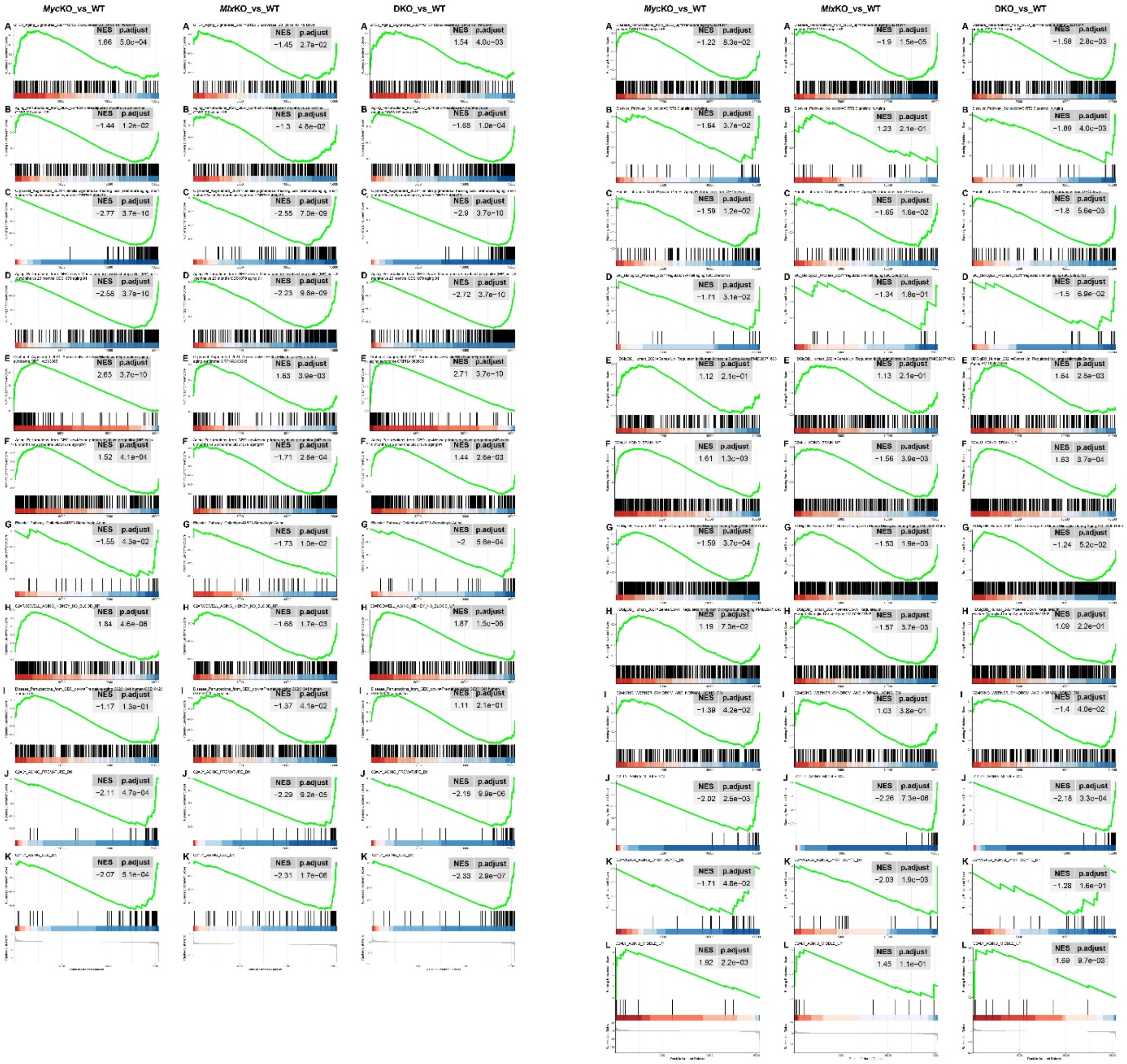

Figure S6. GSEA plots for gene sets involved in aging. Some of these gene sets are also displayed in Fig. 5D as ridgeline plots.

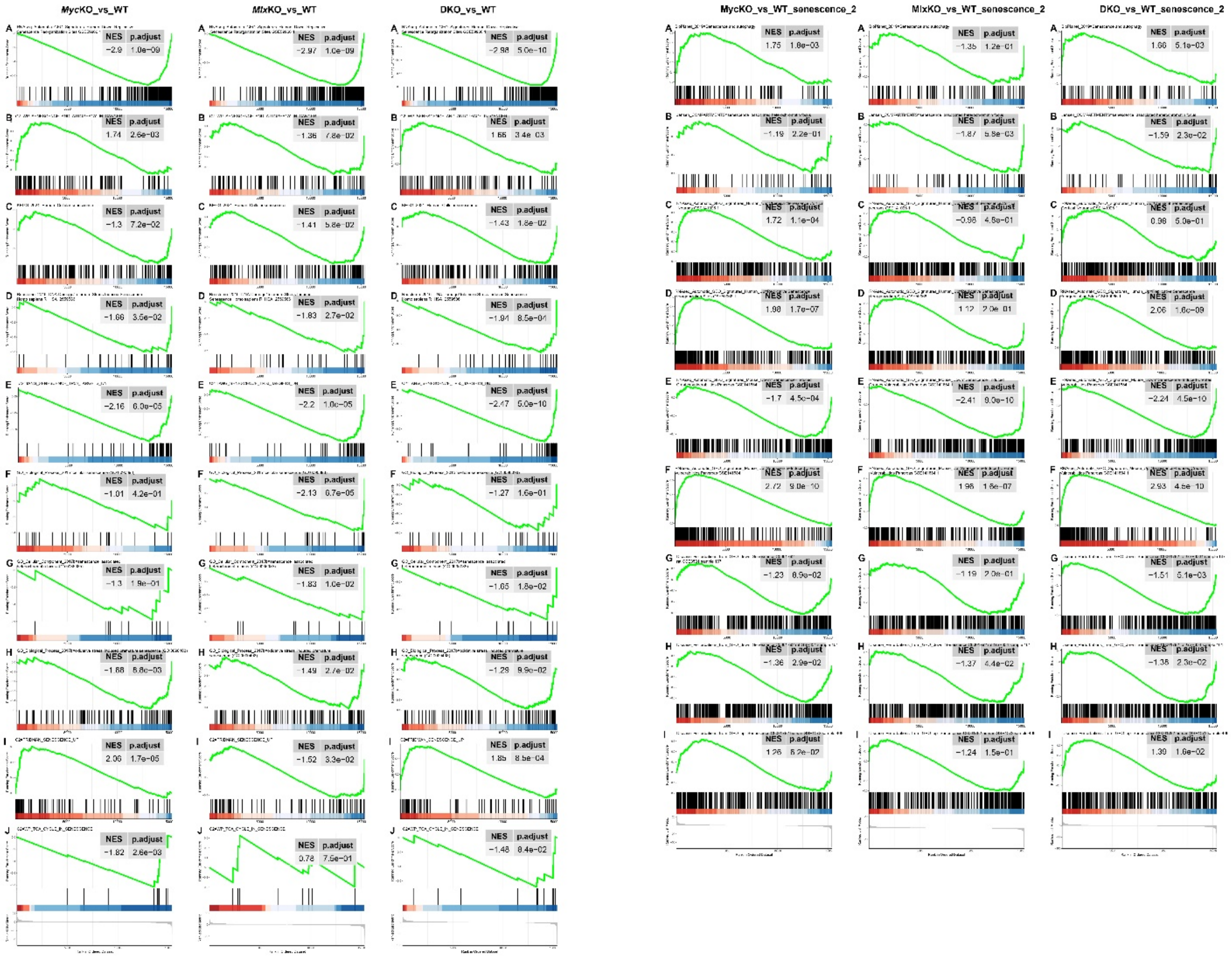

Figure S7. GSEA plots for gene sets involved in senescence. Some of these gene sets are also displayed in Fig. 5D as ridgeline plots.

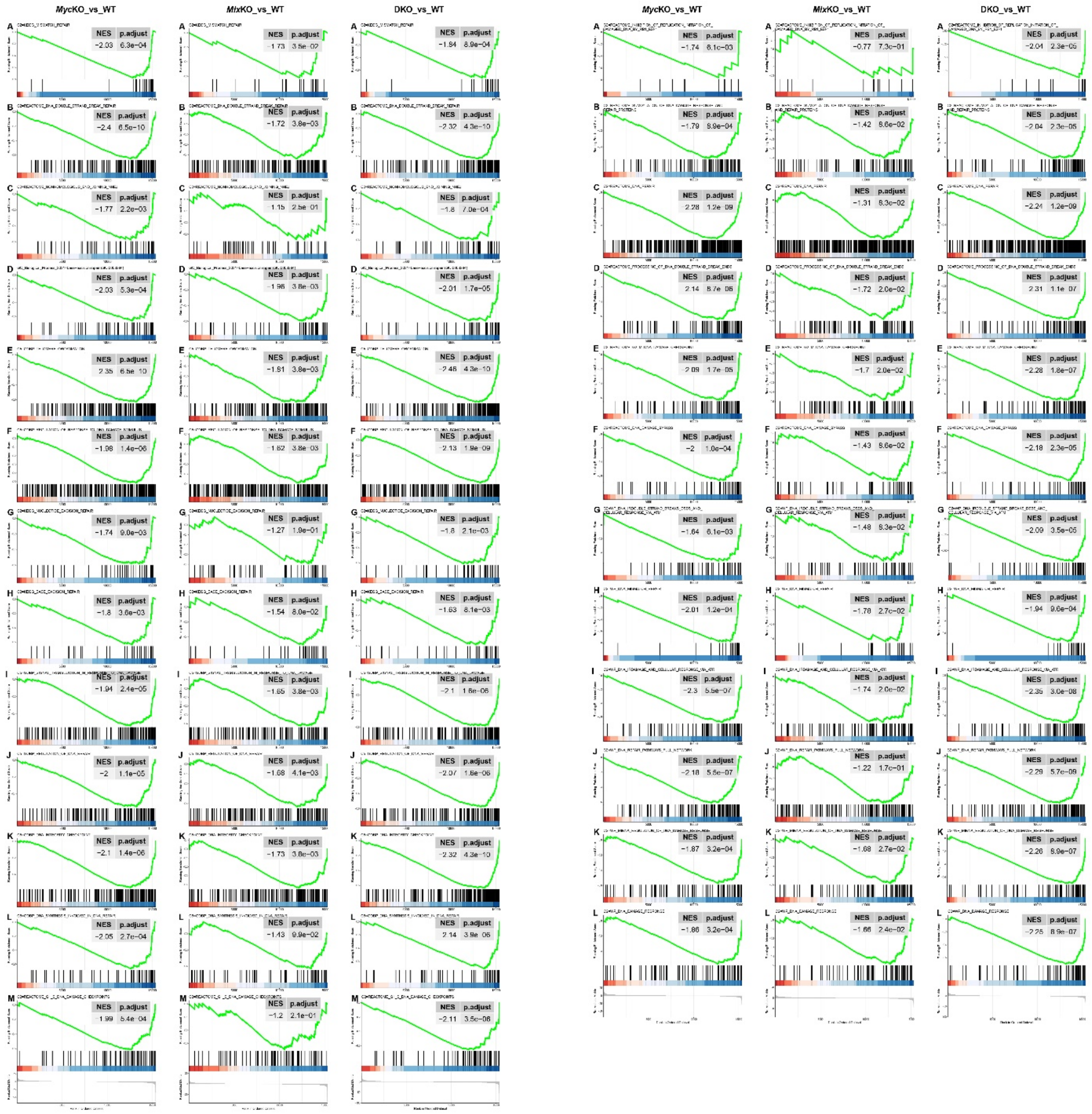

Figure S8. GSEA plots for gene sets involved in DNA damage response/DNA repair. Some of these gene sets are also displayed in Fig. 5D as ridgeline plots.

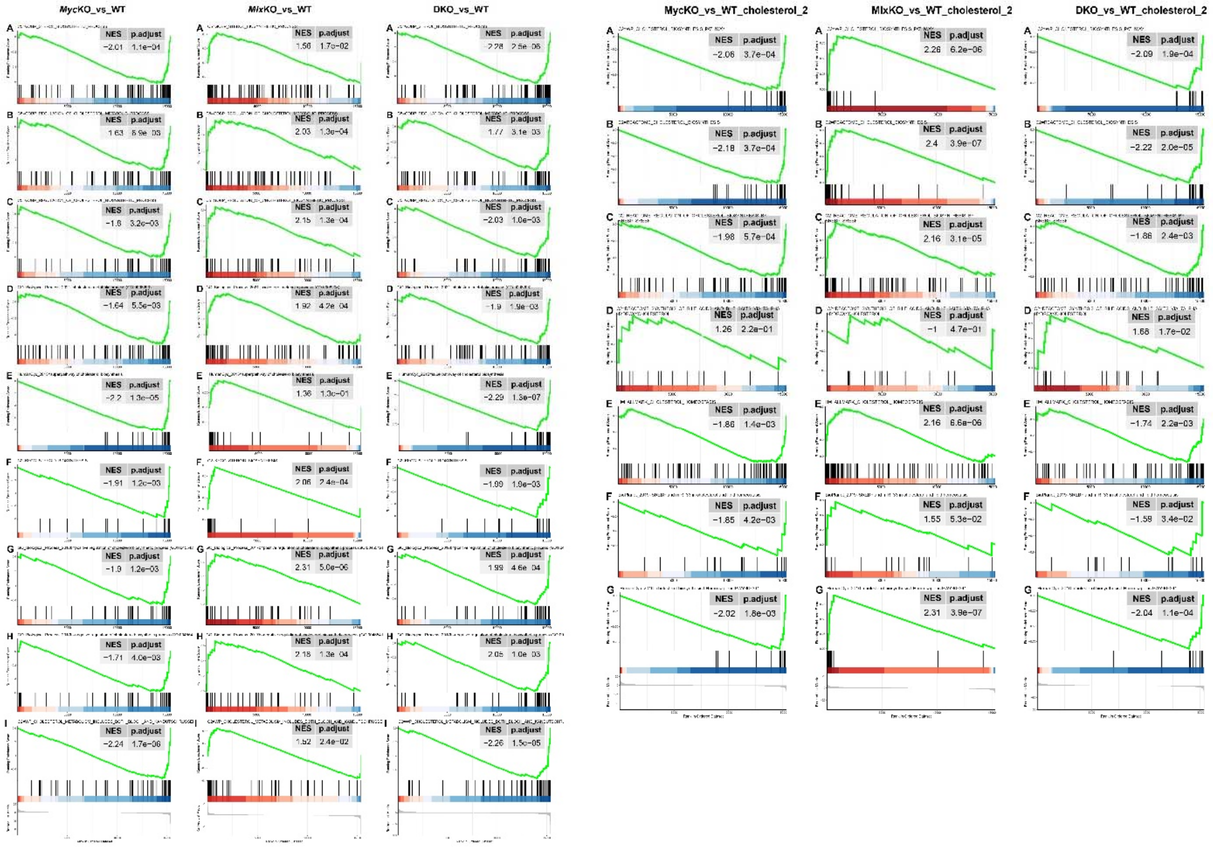

Figure S9. GSEA plots for gene sets involved in cholesterol metabolism. Some of these gene sets are also displayed in Fig. 5D as ridgeline plots.

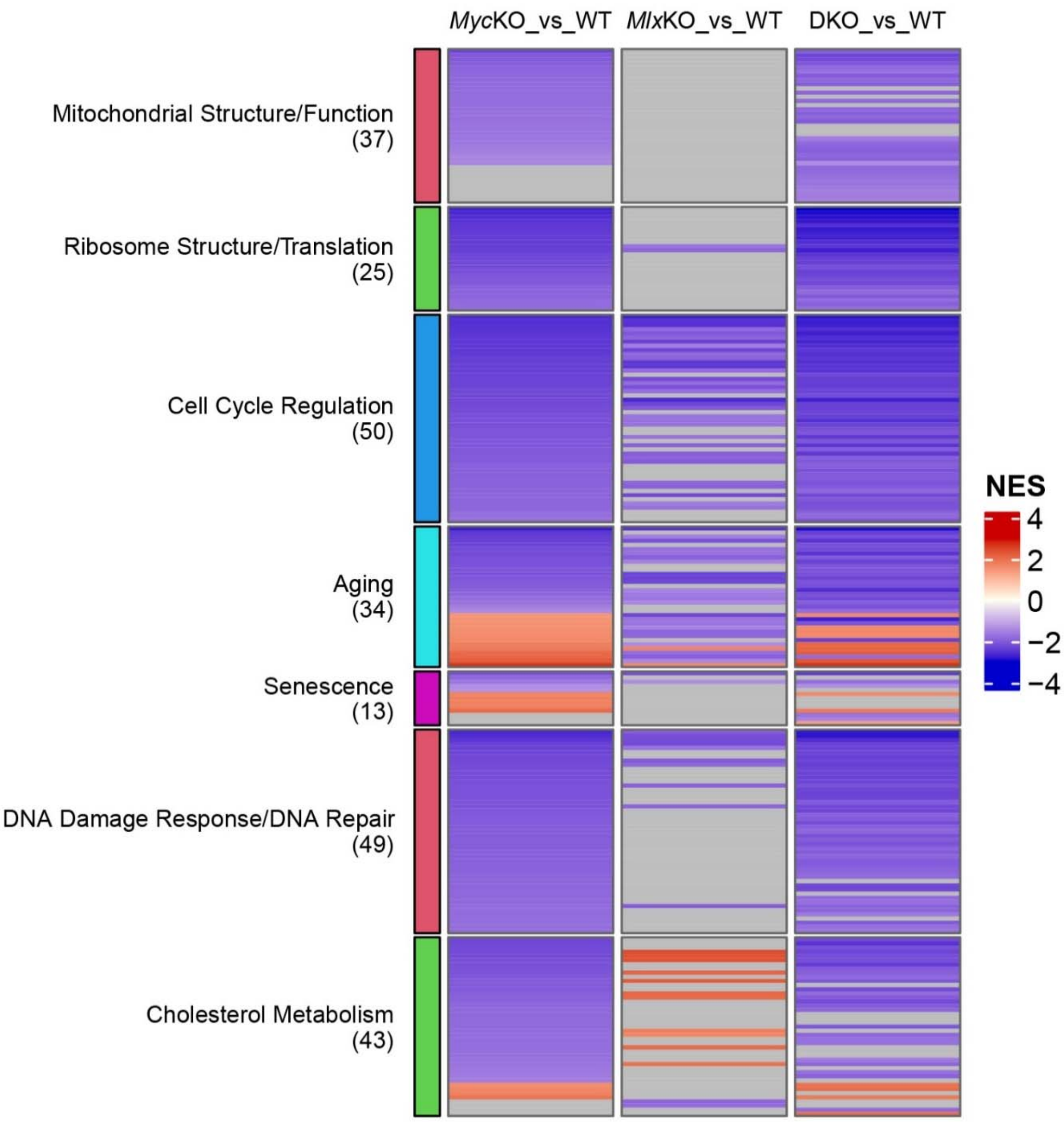

Figure S10. Heat map of the top functional categories of differentially enriched gene sets among each of the three KO MEF lines. Both unbiased and directed GSEA was performed by mining the EnrichR data base (*1, 2*). Each of the top functional categories of enriched genes is indicated to the left of the heat map with number of significantly enriched gene sets contained within the categories indicated beneath. Select members of each group are depicted in the ridgeline blots shown in Fig. 5D and Figs. S3-S9**.**

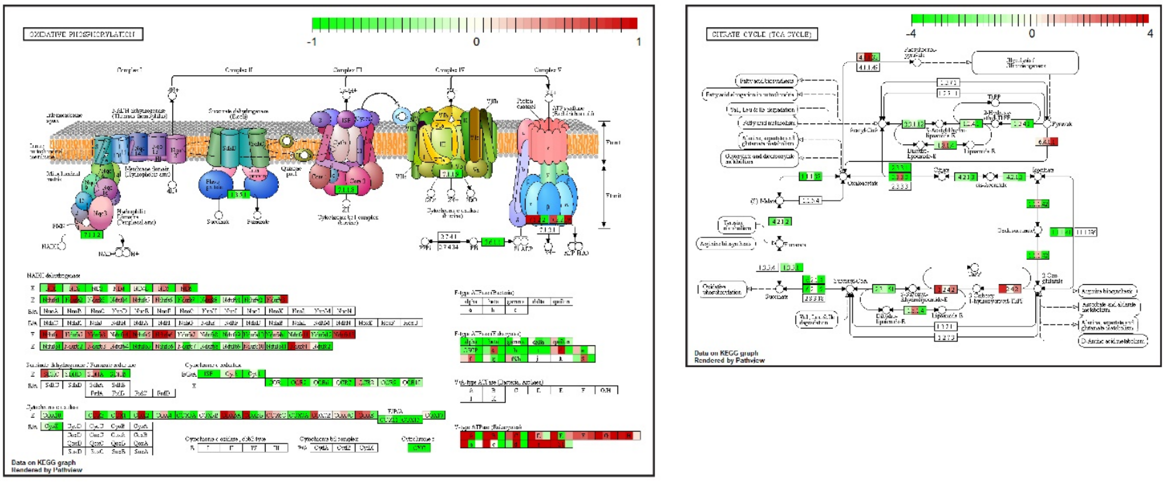

Figure S11. Pathview analysis of individual transcript mean expression levels within the two available "Mitochondrial Structure/Function" KEGG profiles from those depicted in Fig. 5D and Fig. S10. The boxes within which each transcript is enclosed are divided into three segments indicating that transcript's expression relative to that of WT MEFs in *Myc*KO cells (left), *Mlx*KO cells (middle) and DKO cells (right).

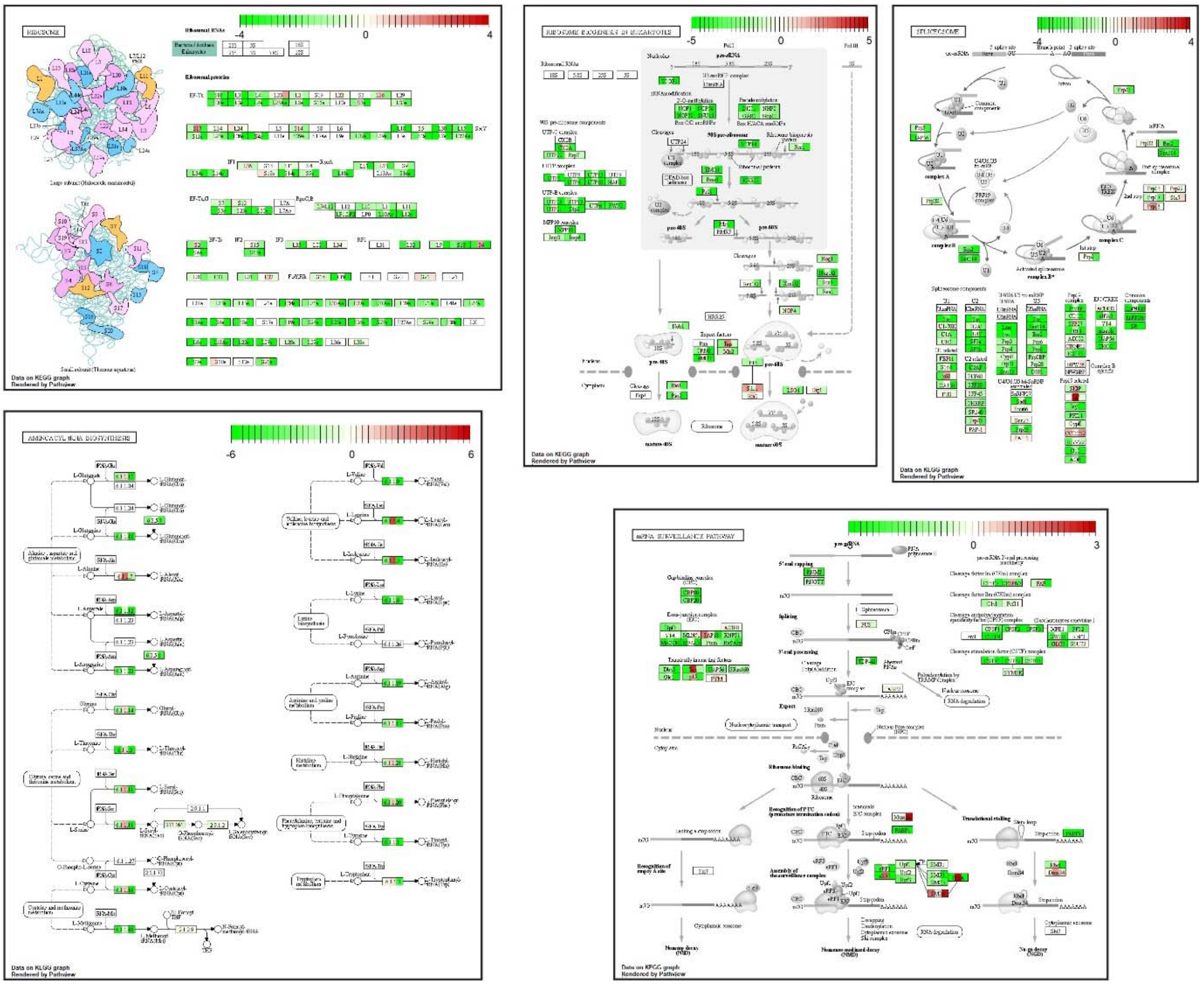

Figure S12. Pathview analysis of individual transcript mean expression levels within the five available "Ribosome Structure/Translation" KEGG profiles from those depicted in Fig. 5D and Fig. S10. The boxes within which each transcript is enclosed are divided into three segments indicating that transcript's expression relative to that of WT MEFs in *Myc*KO cells (left), *Mlx*KO cells (middle) and DKO cells (right).

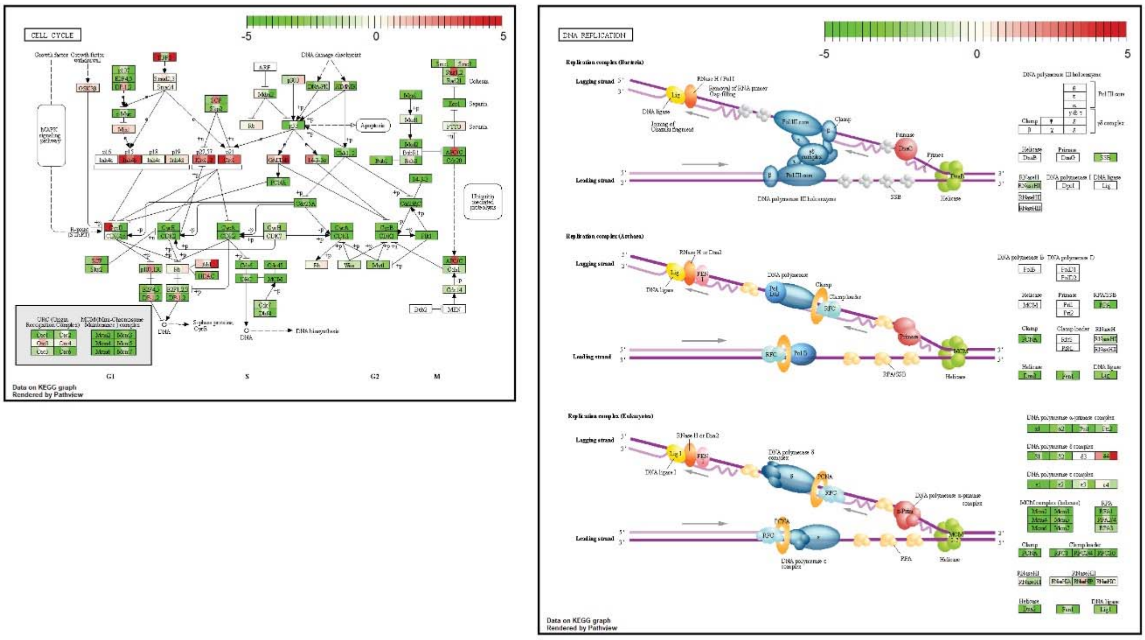

Figure S13. Pathview analysis of individual transcript mean expression levels within the two available "Cell Cycle Regulation" KEGG profiles from those depicted in Fig. 5D and Fig. S10. The boxes within which each transcript is enclosed are divided into three segments indicating that transcript's expression relative to that of WT MEFs in *Myc*KO cells (left), *Mlx*KO cells (middle) and DKO cells (right).

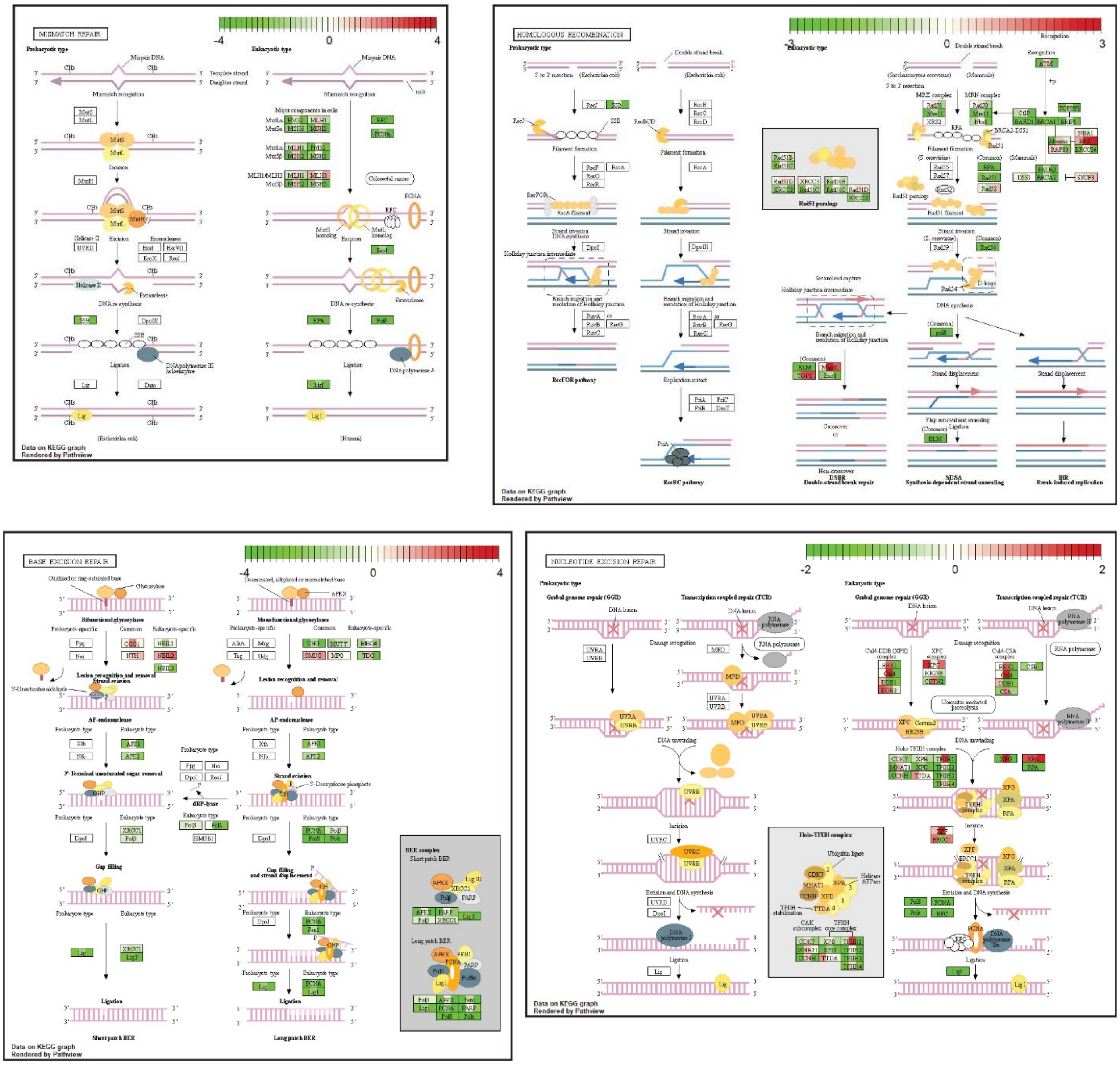

Figure S14. Pathview analysis of individual transcript mean expression levels within the four available "DNA Damage Response/DNA Repair" KEGG profiles from those depicted in Fig. 5D and Fig. S10. The boxes within which each transcript is enclosed are divided into three segments indicating that transcript's expression relative to that of WT MEFs in *Myc*KO cells (left), *Mlx*KO cells (middle) and DKO cells (right).

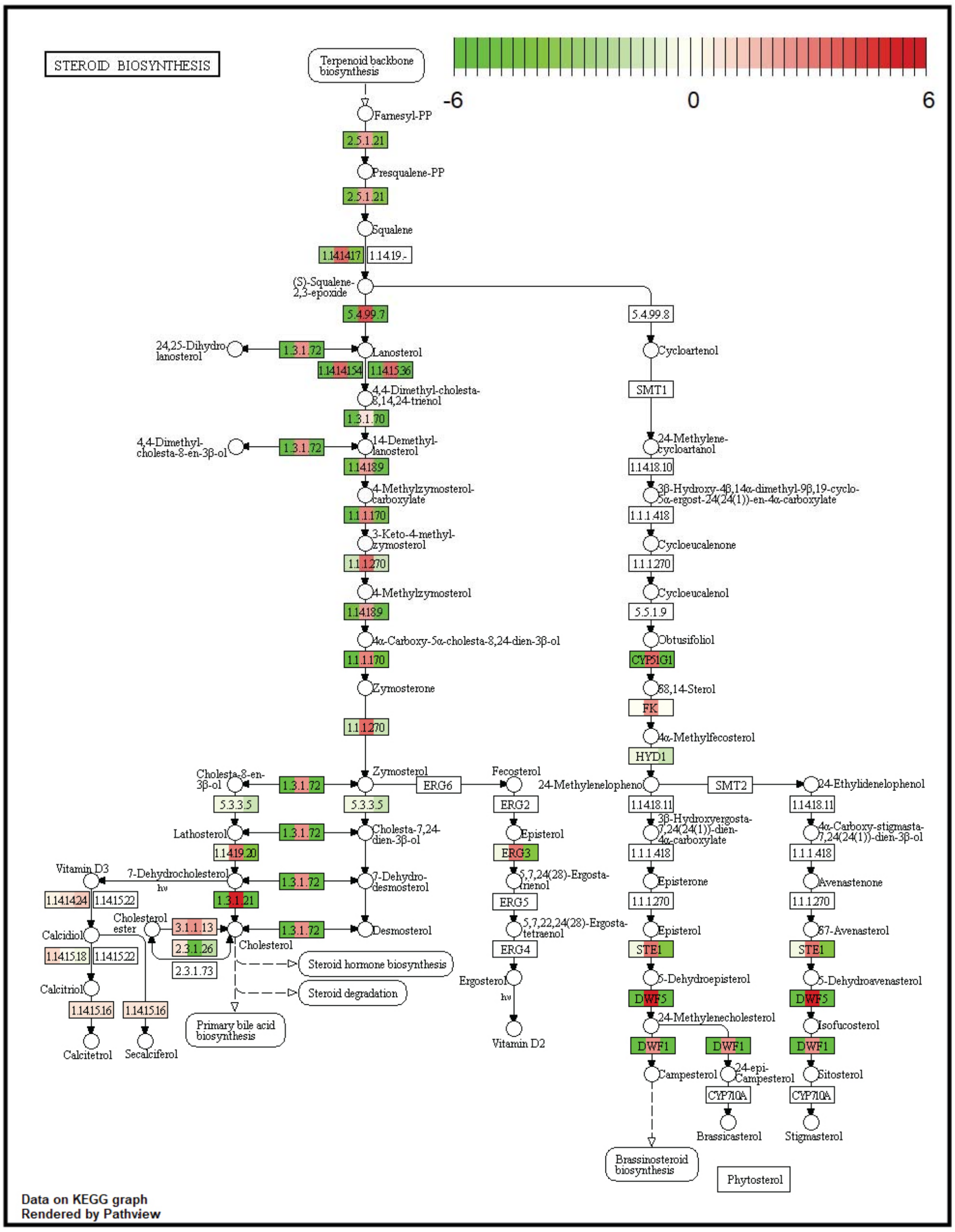

Figure S15. Pathview analysis of individual transcript mean expression levels within the only available "Cholesterol Metabolism" KEGG profile from those depicted in Fig. 5D and Fig. S10. The boxes within which each transcript is enclosed are divided into three segments indicating that transcript's expression relative to that of WT MEFs in *Myc*KO cells (left), *Mlx*KO cells (middle) and DKO cells (right).

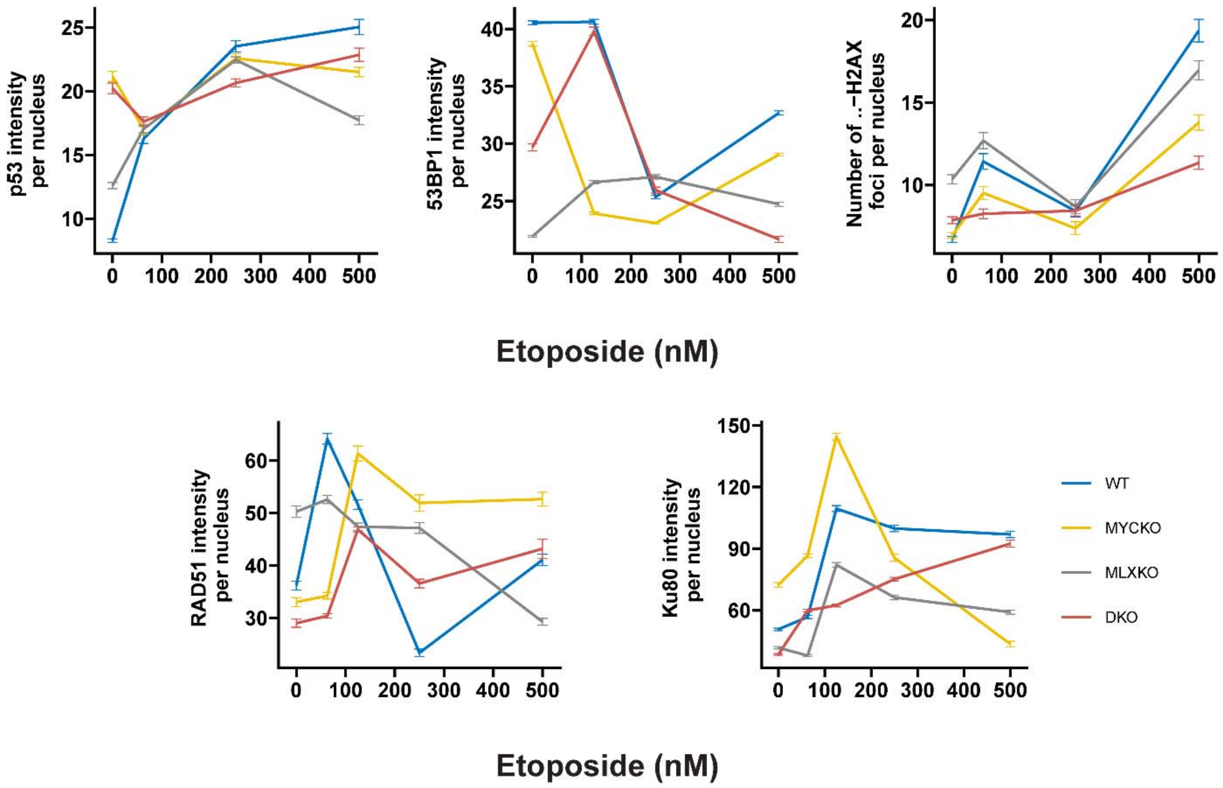

Figure S16. Response of the proteins shown in Fig. 6 to DSBs induced by etoposide. The results from Fig. 6B were re-graphed to better demonstrate the abnormal dose-response kinetics of each MEF cell line. Each point represents the mean intensity or number of foci +/-1 S.E.

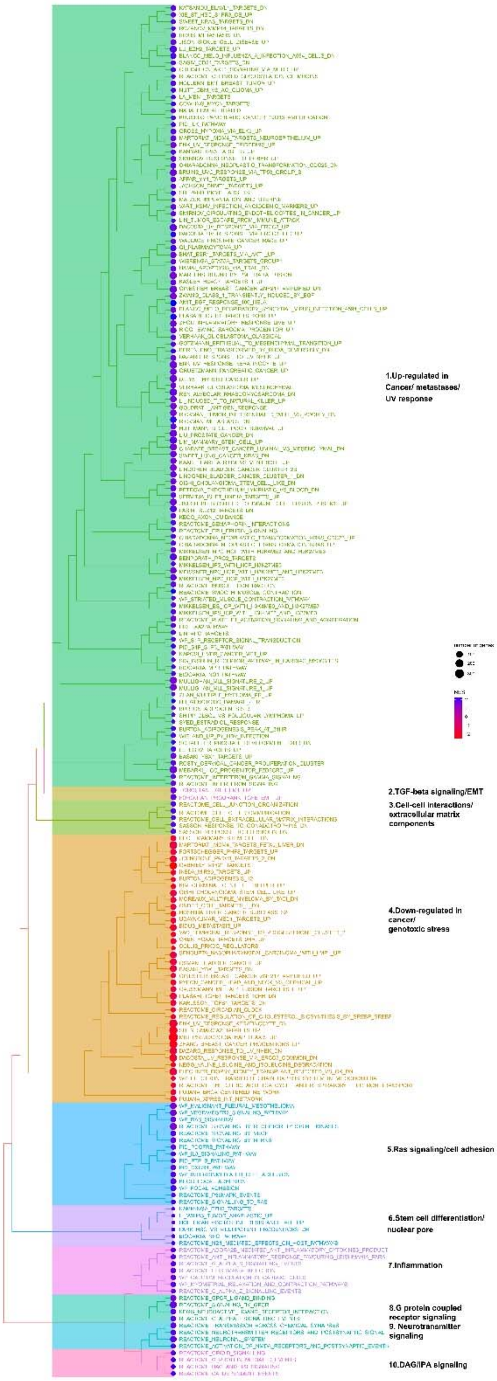

Figure S17. Tree plot for the top ten categories of enriched gene sets in immortalized *Myc*KO vs. WT MEFs. GSEA and data clustering were obtained from the MSigDB C2 data base and analyzed and displayed with the clusterProfiler tool treeplot (https://bioconductor.org/packages/release/bioc/html/clusterProfiler.html). All relevant gene sets with q values <0.05 are shown.

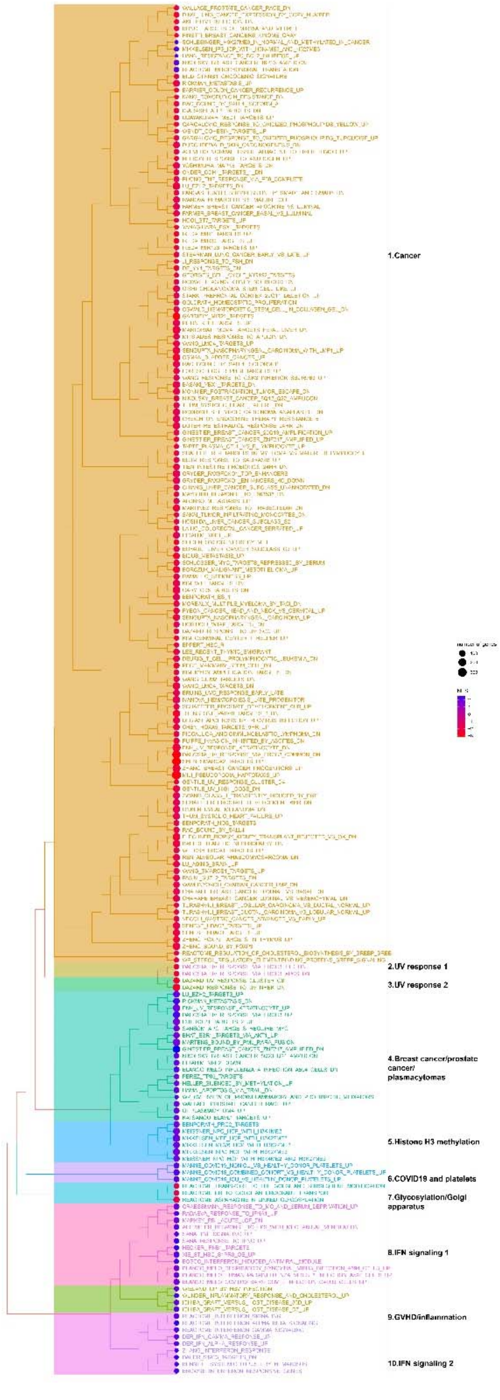

Figure S18. Treeplot for the top ten categories of enriched gene sets in immortalized *Mlx*KO vs. WT MEFs. GSEA and data clustering were obtained from the MSigDB C2 data base and analyzed and displayed with the clusterProfiler tool treeplot (https://bioconductor.org/packages/release/bioc/html/clusterProfiler.html). All relevant gene sets with q values <0.05 are shown.

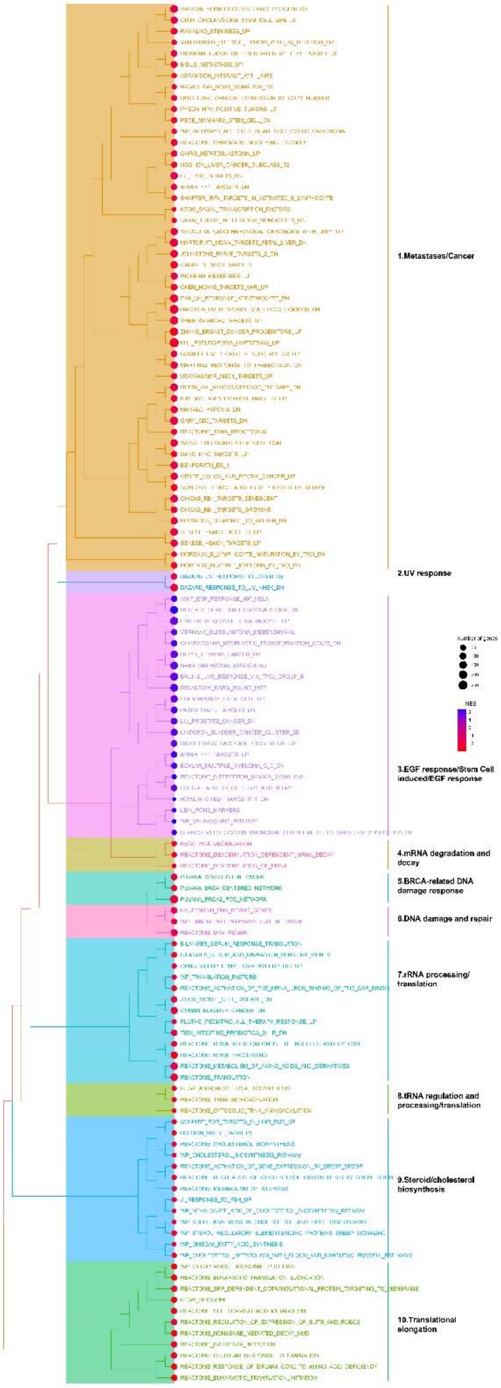

Figure S19. Treeplot for the top ten categories of enriched gene sets in immortalized DKO vs. WT MEFs. GSEA and data clustering were obtained from the MSigDB C2 data base and analyzed and displayed with the clusterProfiler tool treeplot (https://bioconductor.org/packages/release/bioc/html/clusterProfiler.html). All relevant gene sets with q values <0.05 are shown.

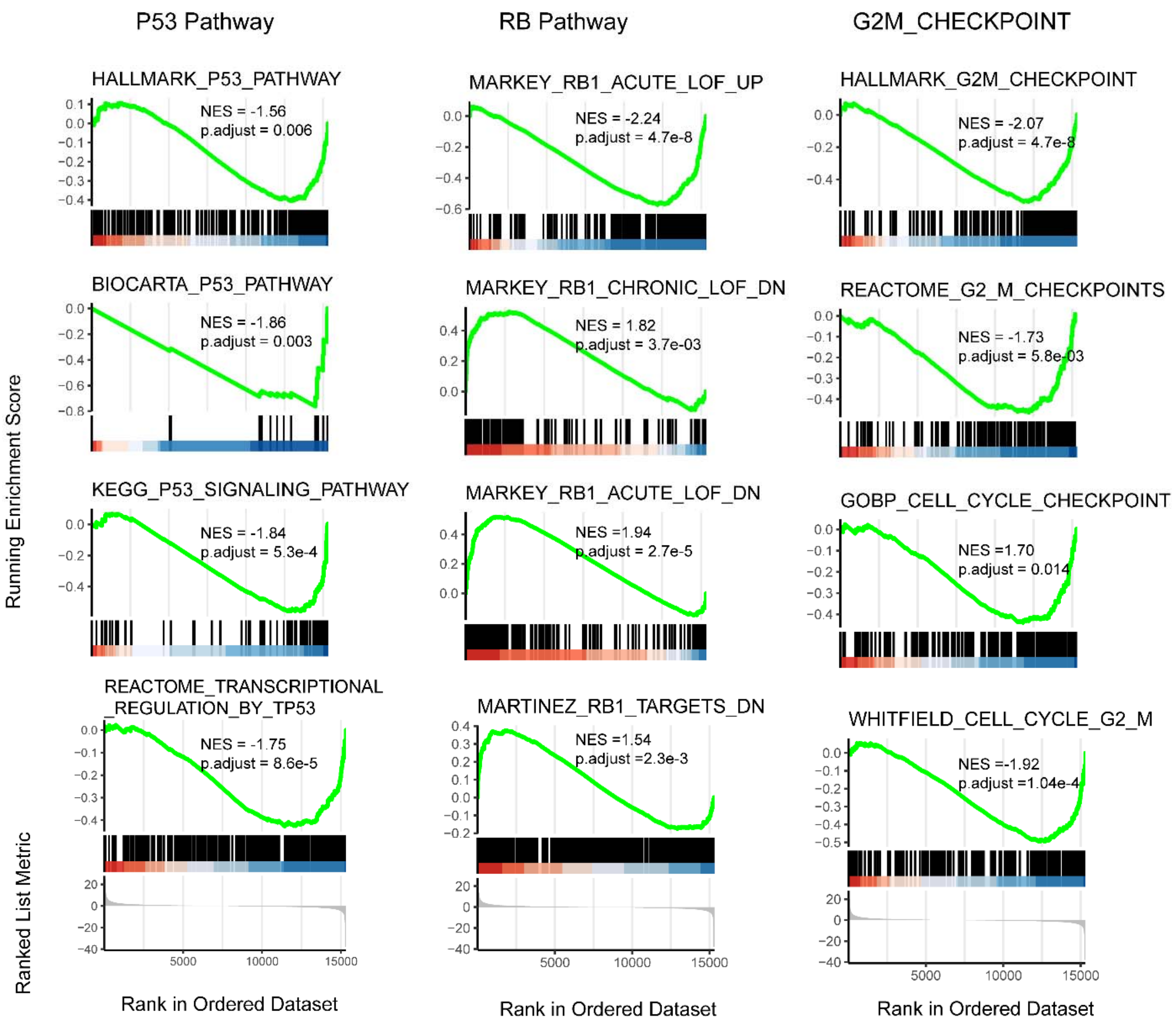

Figure S20. GSEA analysis for primary DKO vs *Myc*KO MEFS using RNAseq data from Fig. 5. Among the top categories of differentially expressed transcripts were those involving the p53 and Rb pathways and G2/M checkpoint blockade, all of which pointed to down-regulation/inhibition in DKO MEFs.

Table S1. List of all antibodies used in the current study.

Name of antigen	Type of antibody	Vendor	Catalog number	Dilution	Purpose
Glut 1	Rabbit mAb	Abcam	Ab115730	1:2000	WB
Glut 4	Mouse mAb	Cell Signaling	2213	1:1000	WB
GAPDH	Mouse mAb	Sigma	G8795	1:10,000	WB
c-Myc	Rabbit mAb	Cell Signaling	13987	1:1000	WB
Mlx	Rabbit mAb	Cell Signaling	85570	1:500	WB
β-Actin	Rabbit mAb	Cell Signaling	3700	1:1000	WB
IgG	HRP-Goat- anti-rabbit	Cell Signaling	7074	1:5000	Secondary for WB
IgG	HRP-Goat- anti-mouse	Cell Signaling	7076	1:10,000	Secondary for WB
γ-H2Ax (pSer139)	Mouse mAb	Millipore	05-636	1:2000	DSB detection (IHC)
Puromycin	Mouse mAb	Millipore	MABE343	1:20,000	WB
RAD51	Rabbit pAb*	Abcam	Ab133534	1:1000	HEJ**(IHC)
53BP1	Rabbit pAb	NovusBio	NB100-304	1:200	DSB detection (IHC)
P53	Rabbit pAb	Abcam	Ab131442	1:1000	DNA damage (IHC)
Ku80	Rabbit mAb	Abcam	Ab80592	1:500	NHEJ***

*pAb=Polyclonal antibody

**HEJ=homologous end-joining

***NHEJ=non-homologous end-joining

Table S2. Dyes used in the current study.

Name of Dye	Used to Detect	Length of Staining	Concentration	Vendor
Propidium Iodide	DNA content	20 min	15 µg/mL	Sigma
Nonyl acridine orange (NAO)	Mitochondrial mass	60 min	0.1 µM	Invitrogen
CM-H2DCFDA	Total ROS	30 min	2.5 µM	Invitrogen
MitoTracker™ Green FM	Mitochondrial Mass	60 min	0.1 µM	Invitrogen
CellEvent™ Senescence Green	SA-β-gal activity	60 min	1:1000	Invitrogen
2-NBDG	Glucose uptake	1-4 hr	100 µM	Invitrogen
BODIPY™ 505/515	Neutral Lipid	30 min	10 µg/mL	Invitrogen
DAPI	Nuclear Stain	10 min	0.2 µg/mL	Sigma
MitoSOX™ Red	Mitochondrial ROS	40min	2.5 µM	ThermoFisher
CellMask™ Orange Actin Tracking Stain	Actin	20min	1:1000	Invitrogen
NucBlue™ Live ReadyProbes™ Reagent (Hoechst 33342)	Nuclear Stain	15min	2 drops/ml	Invitrogen

Table S3. Cell line information from the ENCODE data base used for ChIP studies

File accession	File format	Output type	File assembly	Experiment accession	Assay	Biosample term name	Experiment target	File download URL
**ENCFF780JTP**	bed narrowPeak	IDR thresholded peaks	GRCh38	ENCSR802UCW	TF ChIP-seq	HepG2	MLXIP-human	https://www.encodeproject.org/files/ENCFF780JTP/@@download/ENCFF780JTP.bed.gz
**ENCFF517GVP**	bed narrowPeak	IDR thresholded peaks	GRCh38	ENCSR873LYH	TF ChIP-seq	K562	MLX-human	https://www.encodeproject.org/files/ENCFF517GVP/@@download/ENCFF517GVP.bed.gz
**ENCFF132AJP**	bed narrowPeak	IDR thresholded peaks	GRCh38	ENCSR125DAD	TF ChIP-seq	HepG2	MLX-human	https://www.encodeproject.org/files/ENCFF132AJP/@@download/ENCFF132AJP.bed.gz
**ENCFF869OOK**	bed narrowPeak	IDR thresholded peaks	GRCh38	ENCSR000DMQ	TF ChIP-seq	MCF-7	MYC-human	https://www.encodeproject.org/files/ENCFF869OOK/@@download/ENCFF869OOK.bed.gz
**ENCFF270GMO**	bed narrowPeak	IDR thresholded peaks	GRCh38	ENCSR000EBY	TF ChIP-seq	H1	MYC-human	https://www.encodeproject.org/files/ENCFF270GMO/@@download/ENCFF270GMO.bed.gz
**ENCFF784BWK**	bed narrowPeak	IDR thresholded peaks	GRCh38	ENCSR000DLN	TF ChIP-seq	HeLa-S3	MYC-human	https://www.encodeproject.org/files/ENCFF784BWK/@@download/ENCFF784BWK.bed.gz
**ENCFF239IMY**	bed narrowPeak	IDR thresholded peaks	GRCh38	ENCSR784BVD	TF ChIP-seq	HepG2	MYC-human	https://www.encodeproject.org/files/ENCFF239IMY/@@download/ENCFF239IMY.bed.gz
**ENCFF608CXN**	bed narrowPeak	IDR thresholded peaks	GRCh38	ENCSR000EGJ	TF ChIP-seq	K562	MYC-human	https://www.encodeproject.org/files/ENCFF608CXN/@@download/ENCFF608CXN.bed.gz
**ENCFF735PKA**	bed narrowPeak	IDR thresholded peaks	GRCh38	ENCSR000DLR	TF ChIP-seq	HepG2	MYC-human	https://www.encodeproject.org/files/ENCFF735PKA/@@download/ENCFF735PKA.bed.gz

**ENCFF342ASE**	bed narrowPeak	IDR thresholded peaks	mm10	ENCSR000ERN	TF ChIP-seq	CH12.LX	MYC-mouse	https://www.encodeproject.org/files/ENCFF342ASE/@@download/ENCFF342ASE.bed.gz
**ENCFF459QFK**	bed narrowPeak	IDR thresholded peaks	GRCh38	ENCSR000DLU	TF ChIP-seq	endothelial cell of umbilical vein	MYC-human	https://www.encodeproject.org/files/ENCFF459QFK/@@download/ENCFF459QFK.bed.gz
**ENCFF152JNC**	bed narrowPeak	IDR thresholded peaks	mm10	ENCSR000EUA	TF ChIP-seq	MEL	MYC-mouse	https://www.encodeproject.org/files/ENCFF152JNC/@@download/ENCFF152JNC.bed.gz
**ENCFF385LDF**	bed narrowPeak	IDR thresholded peaks	GRCh38	ENCSR000DMJ	TF ChIP-seq	MCF-7	MYC-human	https://www.encodeproject.org/files/ENCFF385LDF/@@download/ENCFF385LDF.bed.gz
**ENCFF083NGY**	bed narrowPeak	IDR thresholded peaks	GRCh38	ENCSR000DOS	TF ChIP-seq	MCF 10A	MYC-human	https://www.encodeproject.org/files/ENCFF083NGY/@@download/ENCFF083NGY.bed.gz
**ENCFF961HAC**	bed narrowPeak	IDR thresholded peaks	GRCh38	ENCSR000DMP	TF ChIP-seq	MCF-7	MYC-human	https://www.encodeproject.org/files/ENCFF961HAC/@@download/ENCFF961HAC.bed.gz
**ENCFF850VMA**	bed narrowPeak	IDR thresholded peaks	GRCh38	ENCSR000EGS	TF ChIP-seq	K562	MYC-human	https://www.encodeproject.org/files/ENCFF850VMA/@@download/ENCFF850VMA.bed.gz
**ENCFF858ZYN**	bed narrowPeak	IDR thresholded peaks	GRCh38	ENCSR000EHR	TF ChIP-seq	NB4	MYC-human	https://www.encodeproject.org/files/ENCFF858ZYN/@@download/ENCFF858ZYN.bed.gz
**ENCFF598BZD**	bed narrowPeak	IDR thresholded peaks	GRCh38	ENCSR000DYC	TF ChIP-seq	A549	MYC-human	https://www.encodeproject.org/files/ENCFF598BZD/@@download/ENCFF598BZD.bed.gz
**ENCFF377XCI**	bed narrowPeak	IDR thresholded peaks	GRCh38	ENCSR000DMM	TF ChIP-seq	MCF-7	MYC-human	https://www.encodeproject.org/files/ENCFF377XCI/@@download/ENCFF377XCI.bed.gz

**ENCFF792HVZ**	bed narrowPeak	IDR thresholded peaks	GRCh38	ENCSR000DOM	TF ChIP-seq	MCF 10A	MYC-human	https://www.encodeproject.org/files/ENCFF792HVZ/@@download/ENCFF792HVZ.bed.gz
**ENCFF566CTX**	bed narrowPeak	IDR thresholded peaks	GRCh38	ENCSR000DLZ	TF ChIP-seq	K562	MYC-human	https://www.encodeproject.org/files/ENCFF566CTX/@@download/ENCFF566CTX.bed.gz
**ENCFF114VAI**	bed narrowPeak	IDR thresholded peaks	GRCh38	ENCSR744JJU	TF ChIP-seq	K562	MYC-human	https://www.encodeproject.org/files/ENCFF114VAI/@@download/ENCFF114VAI.bed.gz

Table S4. Direct Myc and Mlx target genes from the 7 categories depicted in Fig. 5D and Figs. S3-S9).

Category	Total no. of Transcripts*#	Total no. (%) enriched	Total no. (%)& of direct Myc Targets	Total no. (%)& of direct Mlx Targets	Total no. (%)& of direct Mlx+Myc Targets
Mitochondrial structure/ function	1013	629 (62.1)	497 (79.0)	245 (38.9)	210 (33.3)
Ribosome structure/ function and translation	1338	821 (61.4)	727 (88.5)	362 (44.0)	336 (40.9)
Cell cycle regulation	1066	557 (52.3)	456 (81.8)	219 (39.3)	207 (37.1)
Aging	3014	1544 (51.2)	900 (58.2)	373 (24.1)	321 (20.7)
Senescence	2129	1211 (56.9)	714 (58.9)	314 (25.9)	261 (21.5)
DNA damage response/ DNA repair	878	483 (55.0)	415 (85.9)	195 (40.3)	182 (37.6)
Cholesterol metabolism	239	93 (38.9)	72 (77.4)	29 (31.1)	26 (27.9)
All categories	6880	3714 (54.0)	3714 (54.0)	1120 (30.2)	972 (26.2)

*Transcripts from all available GSEA groups were retrieved from the 7 main categories depicted in Fig. 5D and Figs. S3-S9. 38.9-62.1% of these were found to be enriched. The proximal region of the genes encoding these transcripts (-2.5-+2.5 kb relative to the transcription start site) were then searched in the ENCODE data based to identify documented direct binding sites for Myc and / or Mlx.

&Relative to total no. of enriched targets

#Unique transcripts only; transcripts appearing more than once in any category were counted only once.
